# An update on the impact of pre-transplant transfusions and allosensitization on time to renal transplant and on allograft survival

**DOI:** 10.1186/1471-2369-14-217

**Published:** 2013-10-10

**Authors:** Juan C Scornik, Jonathan S Bromberg, Douglas J Norman, Mayank Bhanderi, Matthew Gitlin, Jeffrey Petersen

**Affiliations:** 1 Department of Pathology, College of Medicine, University of Florida, Gainesville, FL, USA; 2 School of Medicine, University of Maryland, Baltimore, MD, USA; 3 Oregon Health & Science University, Portland, OR, USA; 4 HERON Health, Chandigarh, India; 5 Amgen Inc, Thousand Oaks, CA, USA

**Keywords:** Transfusion, Allosensitization, Renal transplant, Graft survival

## Abstract

**Background:**

Blood transfusions have the potential to improve graft survival, induce sensitization, and transmit infections. Current clinical practice is to minimize transfusions in renal transplantation candidates, but it is unclear if the evidence continues to support pre-transplant transfusion avoidance. Changes in the Medicare prospective payment system may increase transfusion rates. Thus there is a need to re-evaluate the literature to improve the management options for renal transplant candidates.

**Methods:**

A review applying a systematic approach and conducted using MEDLINE^®^, Embase^®^, and the Cochrane Library for English-language publications (timeframe: 01/1984–03/2011) captured 180 studies and data from publically available registries and assessed the impact of transfusions on allosensitization and graft survival, and the impact of allosensitization on graft survival and wait time.

**Results:**

Blood transfusions continued to be a major cause of allosensitization, with allosensitization associated with increased rejection and graft loss, and longer wait times to transplantation. Although older studies showed a beneficial effect of transfusion on graft survival, this benefit has largely disappeared in the post-cyclosporine era due to improved graft outcomes with current practice. Recent data suggested that it may be the donor-specific antibody component of allosensitization that carried the risk to graft outcomes.

**Conclusions:**

Results of this review indicated that avoiding transfusions whenever possible is a sound management option that could prevent detrimental effects in patients awaiting kidney transplantation.

## Background

During the early 1980s, many transplant professionals administered transfusions prior to renal transplant to patients to improve renal graft survival [1]. A decade later few continued to do so, as the risk of sensitization, possible transmission of infection, and improved transplantation outcomes without pre-transplant transfusions did not justify pre-transplant transfusions [2].

The need for red blood cell transfusions for patients with anemia waiting for renal transplantation also decreased with the introduction of erythropoiesis-stimulating agents (ESAs), which are now routinely used in non-emergent situations [3]. However, changes in the Medicare prospective payment system for end-stage renal disease introduced in 2011 [4,5] may increase the use of transfusions. Recent data from the United States Renal Data System (USRDS) reported that the percentage of patients who received at least 1 transfusion increased from 2.4% to 3.0%, a relative increase in transfusion rates of 24% over a 1-year period to September 2011 [6].

A recent Agency for Healthcare Research and Quality (AHRQ) review [7,8] suggested that pre-transplant transfusion resulted in a neutral to beneficial effect on graft rejection, graft survival, and patient survival compared with no transfusion. However, these benefits were reported mostly before the introduction of modern immunosuppressive drugs and solid phase technology to measure sensitization, and the authors acknowledged that strength of the evidence was low. Thus, the evidence for pre-transplant patient management needs re-evaluation to assist patient management.

This study aimed to review the literature and publically available registry data to determine the relationships among pre-transplant transfusion, allosensitization, graft outcomes, and wait time, focusing on the data most relevant to current practice.

## Methods

A literature review using a systematic approach was conducted with the HERON Systematic Review Database, a bespoke structured query language-based internet database. In addition, data were extracted from publically available registry databases. The objectives were to directly assess the impact of pre-transplant transfusions on allosensitization (objective 1) and graft outcomes (objective 2), and the impact of the resulting allosensitization on graft outcomes (objective 3) and wait time (objective 4).

### Data sources

MEDLINE^®^, Embase^®^, and the Cochrane Library were searched for English-language publications. For objectives 1 and 2 (transfusion), a timeframe from January 1, 1984 to March 23, 2011 was used because of the considerable change in pre-transplant transfusion policy after the FDA approval of cyclosporine as an immunosuppressant in November 1983 [9]. For objectives 3 and 4 (allosensitization), a timeframe from January 1, 2001 to March 23, 2011 was used because data collection was limited to the most relevant and current studies, given that the recent technological developments in allosensitization measurement would have reduced the applicability of older publications to current clinical practice.

Six transplantation registries were searched for patient-level data: the Australia and New Zealand Dialysis and Transplant Registry (ANZDATA; http://www.anzdata.org.au/v1/index.html), Collaborative Transplant Study (CTS; http://www.ctstransplant.org/public/publications.jsp), United Network for Organ Sharing (UNOS; http://www.unos.org/), USRDS (http://www.usrds.org/), American Society of Transplantation (AST; http://www.a-s-t.org/), and American Association of Blood Banks (AABB; http://www.aabb.org/Pages/Homepage.aspx). The AST and AABB did not yield any data for inclusion in this study.

### Search strategy for literature review

The search strategy involved 5 primary facets (comprised of medical subject headings [MeSH], keywords, and Emtree terms [used to index the Embase database], as appropriate) that were combined to answer the different study objectives; these facets focused on organ, antibody, transfusion, transplantation, and outcome. The disease area of interest was kidney transplantation. The use of an organ facet rather than a specific kidney facet ensured that studies enrolling potential transplant patients who did not proceed to transplant were captured. An outcome facet was required to identify studies where allosensitization was associated with wait time and/or renal allograft survival. A search sample is provided in Additional file 1.

### Study eligibility, selection, and data extraction for literature review

All study designs were included. Studies involving pre- and post-renal transplant patients with chronic kidney disease, end-stage renal disease, or dialysis, who had received transfusions of leukoreduced or non-leukoreduced red blood cell units, as well as whole blood, were included. There were no restrictions on age, gender, country, or race. Animal studies, laboratory and validation studies of clinical assays, case studies, conference abstracts and publication, review and editorial articles, and non-English publications were excluded.

Bibliographic details and abstracts of all citations identified by the literature search were downloaded into the HERON Systematic Review Database. A team of reviewers independently determined the eligibility of each publication by applying the eligibility criteria to each citation. Citations and then full-text papers were screened by 2 independent reviewers, and any discrepancies between reviewers were reconciled by a third independent reviewer.

Eligible studies were extracted to pre-defined data extraction grids. Where > 1 publication was identified that described a single trial, data were compiled into a single entry to avoid double-counting of patients. Data extracted included study design, patient population characteristics, degree of sensitization as measured by cytotoxicity (panel reactive antibodies [PRA]; %) or solid phase techniques, degree of allosensitization as measured by presence of donor-specific antibodies (DSAs; %), rates of pre-transplant mortality in allosensitized patients (%), median wait time to transplant (days, months, years), patients awaiting transplantation at specific time points (%), graft survival rate (%; at 3, 6, 12, 24, 36, 60, 120, and 360 months), patient survival rate (%; at 3, 6, 12, 24, 36, 60, 120, and 360 months), and incidence of acute, chronic, and antibody-mediated rejection (%).

Following data extraction, additional criteria were applied to the included studies to identify the most appropriate data for each objective. Non-peer-reviewed publications were excluded, as they were assumed to contain less reliable data than peer-reviewed publications. In addition, studies comparing different types of transfusion (e.g. donor-specific vs. random) were excluded as these studies do not directly answer the review objectives (comparing transfusion vs. no transfusion). Similarly, studies presenting data in an unsuitable format (e.g. no numerical data, data for one patient group only, or data for time points different from those of interest in the review) were excluded from the analysis as they could not be shown in graphical format for comparison with other studies.

### Data analysis

The quality of the studies included in the literature review was assessed using the Downs and Black checklist [10]. *P*-values and statistics are unadjusted and reported as stated in their original publications except where indicated. Results are presented in percentages for specific populations of patients (e.g. transfused and sensitized, transfused and non-sensitized). When not reported, these percentages were calculated from the study data. In studies where patients could be grouped in 1 category (e.g. sensitized, non-sensitized), patient numbers were pooled to calculate percentages. Where appropriate, all time points with data available were plotted; in some cases, key time points only were plotted (e.g. 12 months) due to a lack of data at other time points.

Qualitative analysis was conducted, including expert review by specialists in renal transplantation. Quantitative pooled analysis was not considered appropriate due to the heterogeneity in the study designs and patient populations.

## Results

**Figure 1:**
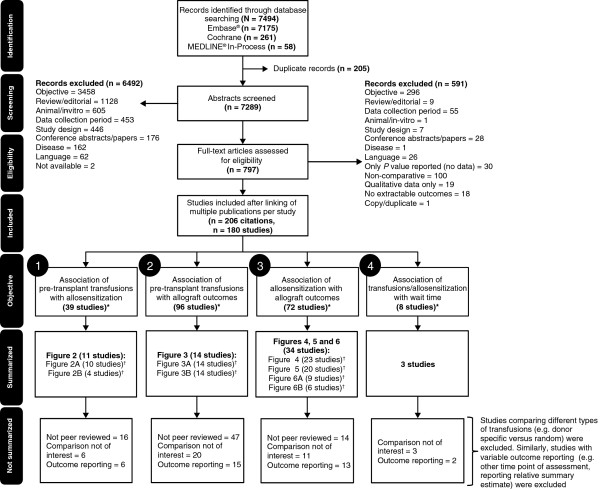
**PRISMA diagram depicting study flow through the review.** *The number of studies does not sum to 180 across all objectives since a study may answer more than one objective. ^†^The number of studies for each figure does not sum to the number of studies summarized since a study may be presented in different figures or multiple parts of a single figure. Not peer-reviewed: studies published in non-peer-reviewed journals (specifically *Clinical Transplants and Transplantation Proceedings*). Comparison not of interest: includes comparing various types or volume of transfusion (e.g. donor specific versus random); Outcome reporting: data for the outcome have not been reported in a format suitable for graphical representation (e.g. outcomes reported for one patient group only, outcome was drawn from a general conclusion in the text, time points were different from those selected for extraction [3, 6, 12, 24, 36, 60, 120, and 360 months], or outcomes were impacted by the different antibody detection techniques used in the study).

A total of 7494 citations were identified, of which 206 citations relating to 180 studies were relevant to the objectives. Of these, 62 studies were summarized (Figure 1). The Downs and Black [10] quality score for these studies ranged from 13 to 22 out of a possible score of 26. Although there is no definitive cut-off for an acceptable score, a recent publication on evidence assessment considered > 14 as acceptable [11]. Using this criteria, majority of the studies were considered to be of acceptable quality, and only 6 were considered to be of poor quality [1,12-16].

### The impact of pre-transplant transfusion on allosensitization

**Figure 2:**
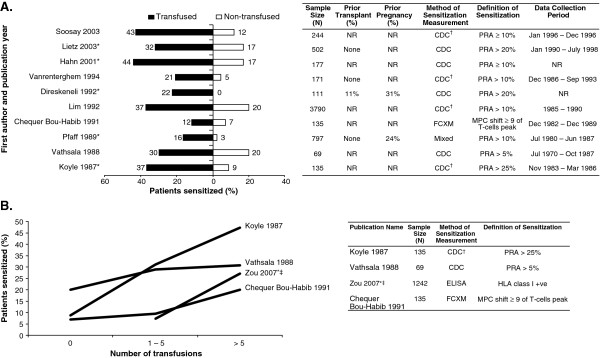
**Impact of pre-transplant transfusion on allosensitization. (A)** Proportion of patients sensitized stratified by pre-transplant transfusion status. **(B)** Risk of allosensitization by number of pre-transplant transfusions. *Significant difference as reported in the original publication; ^†^represents sensitization measurement not explicit, but identified according to the text; ^‡^data reported for 0–5 transfusion vs. > 5 transfusions; values have been rounded to the nearest integer. NOTE: Additional calculation has been performed to allow for comparison between the populations of interest. Therefore, the numbers presented differ from those presented in the primary source publications with the exception of Lim 1992, Direskeneli 1992, and Lietz 2003. CDC: complement-dependent cytotoxicity; ELISA: enzyme-linked immunosorbant assay; FXCM: flow cytometry cross-match; HLA: human leukocyte antigen; Mixed: probable CDC + flow cytometry; MPC: mean peak channel; NR: not reported; PRA: panel reactive antibodies; +ve: positive.

All studies included in the analysis reported a detrimental effect of pre-transplant transfusion on allosensitization (Figure 2): a significant detrimental effect was reported in 6 studies [1,17-21] while a non-significant detrimental effect was reported in the remaining 5 studies [22-26].

In general, the rate of allosensitization was higher in patients receiving transfusion compared with patients not transfused (Figure 2A). Allosensitization was also influenced by the number of pre-transplant transfusions, with an increased number of prior transfusions augmenting the risk of allosensitization (Figure 2B) [19,21,22,26].

The USRDS 2010 annual report confirmed the sensitizing effect of transfusions. Patients receiving transfusions had a higher risk of sensitization compared with those not receiving transfusions. Parous females receiving pre-transplant transfusions were at increased risk of sensitization compared with non-parous females (odds ratio [OR] for sensitization with transfusion compared with no transfusion for parous females vs. non-parous females [PRA level]: 1.43 vs. 1.03 [> 0%], 1.44 vs. 1.08 [≥ 10%], 1.51 vs. 1.10 [≥ 20%], 1.61 vs. 1.12 [≥ 50%], and 1.76 vs. 1.26 [≥ 80%]) [27]. Males had a higher risk of sensitization after pre-transplant transfusion compared with non-parous females (OR for sensitization with transfusion compared with no transfusion for males vs. non-parous females [PRA level]: 1.17 vs. 1.03 [> 0%], 1.43 vs. 1.08 [≥ 10%], 1.59 vs. 1.10 [≥ 20%], 1.86 vs. 1.12 [≥ 50%], and 2.38 vs. 1.26 [≥ 80%]) [27], although the influence of previous transplants was not assessed.

### The impact of pre-transplant transfusions on allograft outcomes

**Figure 3:**
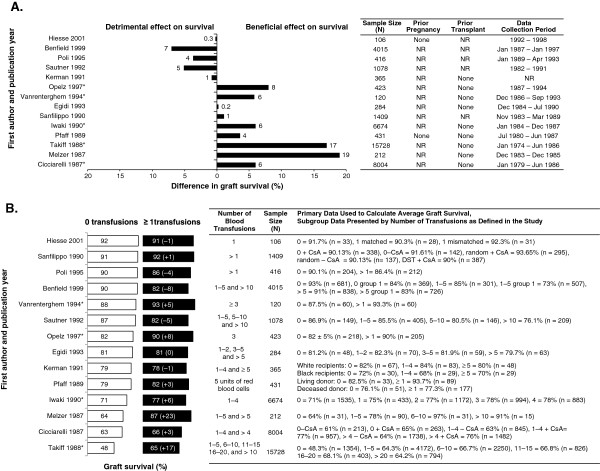
**Graft survival at 12 months. (A)** Difference between transfused and non-transfused patients. **(B)** Number of pre-transfusions. *Significant difference as reported in the original publication; values have been rounded to the nearest integer, unless < 1. NOTE: Additional calculation has been performed to allow for comparison between the populations of interest. Therefore, the numbers presented differ from those presented in the primary source publications with the exception of Poli 1995 and Opelz 1997. NR: not reported; n = sample size with reported outcome; CsA: cyclosporin A; DST: donor-specific transfusion.

Most studies reported a beneficial effect of pre-transplant transfusion on graft survival at 12 months (Figure 3A): 5 studies reported a significant beneficial effect [25,28-31], and 4 studies a non-significant beneficial effect [1,32-34]. In contrast, a non-significant detrimental effect of pre-transplant transfusions on graft survival was reported in 5 studies [2,35-38]. A possible reason for the conflict between studies is the higher 1-year graft survival in patients without pre-transplant transfusion, typical of current outcomes, compared with older reports. Figure 3B indicates that the beneficial effect of pre-transplant transfusions was observed when graft survival rates in patients without pre-transplant transfusion were low, with the difference in graft survival in patients receiving or not receiving pre-transplant transfusions becoming less apparent as graft outcomes improved over time.

### The impact of allosensitization on allograft outcomes

**Figure 4:**
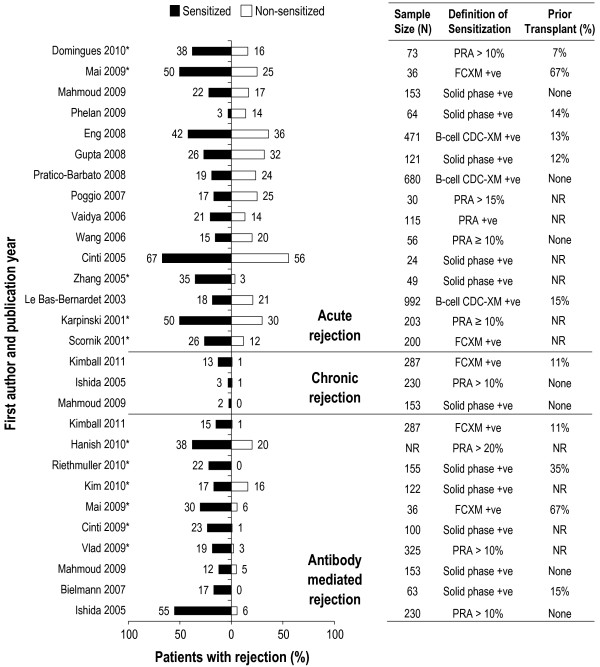
**Impact of allosensitization on graft rejection following kidney transplantation measured by acute rejection, chronic rejection, and antibody-mediated rejection.** *Significant difference as reported in the original publication; values have been rounded to the nearest integer. NOTE: Additional calculation has been performed to allow comparison of populations of interest. Therefore, the numbers differ from the primary publications with the exception of Mai 2009, Eng 2008, Pratico-Barbato 2008, Wang 2006, Le Bas-Bernardet 2003, Karpinski 2001, Scornik 2001, Hanish 2010, Riethmuller 2010, Cinti 2009, and Vlad 2009. CDC: complement-dependent cytotoxicity; CDC-XM: complement-dependent cytotoxicity cross-match; FCXM: flow cytometry cross-match; NR: not reported; PRA: panel reactive antibodies; +ve: positive.

Most studies reported a detrimental effect of allosensitization on graft rejection: 9 studies reported a significant detrimental effect [13,14,39-45], and 7 studies reported a non-significant detrimental effect [12,15,16,46-49] (Figure 4). In contrast, 6 studies reported a non-detrimental effect of allosensitization on graft rejection [50-55]. Of these, 2 restricted the analysis to B cell antibodies [51,54].

**Figure 5:**
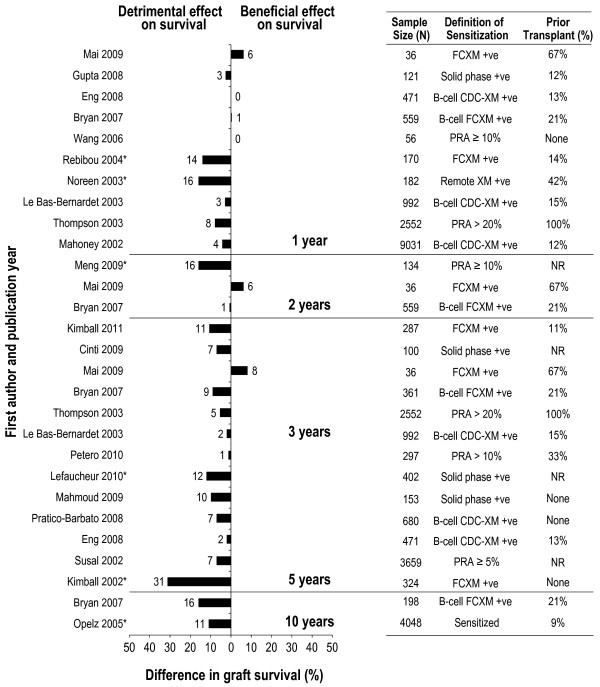
**Relationship between allosensitization and graft survival at 1 year, 2 years, 3 years, 5 years, and 10 years.** *Significant difference as reported in the original publication; values have been rounded to the nearest integer. NOTE: Additional calculation has been performed to allow for comparison between the populations of interest. Therefore, the numbers presented differ from those presented in the primary source publications for Gupta 2008, Bryan 2007, Thompson 2003, Kimball 2011, Petero 2010, Lefaucheur 2010, Susal 2002, Kimball 2002, and Opelz 2005. CDC: complement-dependent cytotoxicity; CDC-XM: complement-dependent cytotoxicity cross-match; FCXM: flow cytometry cross-match; NR: not reported; PRA: panel reactive antibodies; +ve: positive.

When considering graft survival, 6 studies reported a significant detrimental effect of allosensitization on graft survival [56-61] and 12 studies reported a non-significant detrimental effect [13,16,47,48,50,51,54,57],[62-65] (Figure 5). In contrast, 2 studies reported a non-detrimental impact of allosensitization on graft survival. Two additional studies found no effect of B-cell antibodies on graft survival [47,62].

Overall, allosensitization was linked with higher rates of graft rejection and lower rates of graft survival compared with non-sensitized patients. In a recently published study, the incidence of rejection was significantly higher in patients with sensitization (PRA ≥ 10%; 58.8% patients with rejection vs. 23.3% patients without rejection) than in patients without sensitization (PRA < 10%; 35.3% patients with rejection vs. 76.8% patients without rejection) [14]. Similarly, a recent study reported lower graft survival in sensitized patients compared with non-sensitized patients (1 year: 85% vs. 95%; 3 years: 75% vs. 94%; 8 years: 60.6% vs. 83%; *P* < 0.001) [57]. One study found no significant differences in either rejection or graft survival in sensitized patients compared with non-sensitized patients [54].

**Figure 6:**
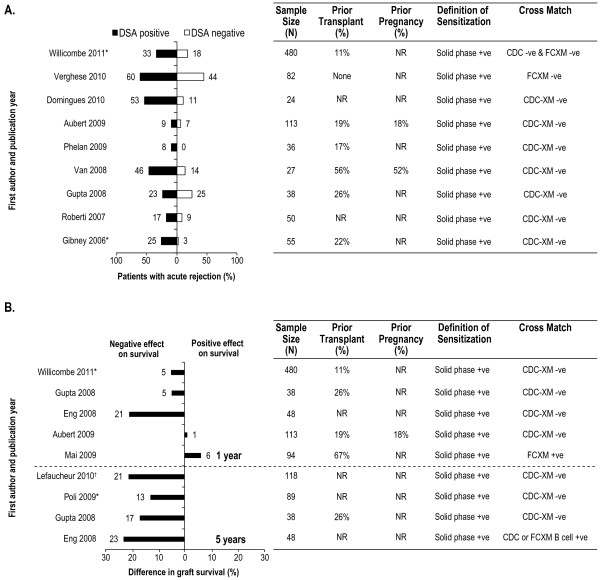
**Impact of donor specific antibodies on graft rejection and survival. (A)** Graft rejection. **(B)** Graft survival. *Significant difference as reported in the original publication; ^†^death censored graft survival reported; values have been rounded to the nearest integer. NOTE: Additional calculation has been performed to allow for comparison between the populations of interest. Therefore, the numbers presented differ from those presented in the primary source publications for Domingues 2010, Van 2008, Gupta 2008, and Lefaucheur 2010. CDC: complement-dependent cytotoxicity; CDC-XM: complement-dependent cytotoxicity cross-match; FCXM: flow cytometry cross-match; NR: not reported; -ve: negative; +ve: positive.

Considering the impact of DSAs, 8 of the 9 studies reported that the presence of DSAs was associated with more acute graft rejections [14,50,52,66-71], with 2 studies reporting significant differences between groups [67,71] (Figure 6A). In addition, DSAs were associated with lower graft survival in 5 out of 6 studies investigating this relationship [47,50,52,57,71], with 2 studies reporting significant differences between groups [71,72] (Figure 6B). In contrast, no differences were observed in 3 studies, either with acute rejection [50] or graft survival [41,66].

In the USRDS 2010 annual report, the risk of graft failure was higher in allosensitized versus non-allosensitized patients (hazard ratio for PRA levels of 0%: 1.0; 1%–9%: 1.08; 10%–79%: 1.21; ≥ 80%: 1.41) [27], while the UNOS/Scientific Registry of Transplant Recipients (UNOS/SRTR) 2010 annual report showed a decrease in long-term graft survival (5 and 10 years) with increased PRA levels (i.e. increase in allosensitization), irrespective of the donor type (living or deceased) [73]. From the 2012 Organ Procurement and Transplantation Network/Health Resources and Services Administration (OPTN/HRSA) registry data, Kaplan-Meier estimates of survival rates for kidney transplants performed between 1997 and 2004 reported that graft survival decreased with increased PRA levels (patients with 0–9% PRA: survival rate of 91.8%, 82.5%, and 72.2% for 1 year, 3 years, and 5 years, respectively; patients with 10%–79% PRA: survival rate of 89.6%, 77.4%, and 65.7% for 1, 3, and 5 years, respectively; patients with ≥ 80% PRA: survival rate of 88.4%, 74.6%, and 61.9% for 1, 3, and 5 years, respectively) [74].

### The impact of allosensitization on wait time to transplantation

A detrimental effect of sensitization on wait time was reported in the 3 studies [24,49,75], reporting non-significant increases in wait time in sensitized patients compared with non-sensitized patients. Overall, sensitization was linked with longer wait times compared with non-allosensitized patients.

Data from the USRDS 2010 annual report suggested that, alongside a general increase in the median wait time to transplantation in all patients in the last 20 years, wait time was increased further in patients with allosensitization compared with patients without allosensitization [27]. The USRDS 2011 annual report indicated a trend for increased median wait time to transplantation with increasing allosensitization (PRA levels of 0%: 1.86 years; 1–9%: 1.84 years; 10–79%: 2.09 years; ≥80%: 2.88 years) [76]. Similar trends were observed in the UNOS 2010 annual report; median wait time to transplantation was higher in allosensitized patients (PRA ≥ 10%) compared with non-allosensitized patients, with the longest wait time in patients with PRA levels between 20% and 79% [73].

## Discussion

The pre-transplantation transfusion practice over the last 20 years has been to minimize transfusions because of risks outweighing benefits. Practice may change following the recent changes in payments for the management of end-stage renal disease for Medicare patients [4,5] and data from an AHRQ review [7,8] suggesting that pre-transplant transfusion resulted in a neutral to beneficial effect on graft rejection, graft survival, and patient survival compared with no transfusion. Although the authors of the review acknowledged that the strength of the evidence was low. While literature on pre-transplant transfusions is abundant, the variety of patient characteristics, questions addressed, methods used, data details presented, and general study quality presents a challenge for the assessment of the effects of pre-transplant transfusions on patient outcomes. Our review aimed to address this issue through the systematic identification of high-quality studies (defined by peer review) that assessed the relationship between pre-transplant transfusion and patient outcomes of allosensitization, graft survival, and wait time, and report if these relationships were observed in patient-level registry data.

The knowledge that pre-transplant transfusions can cause human leukocyte antigen (HLA) sensitization dates to the early days of transplantation in the 1960s and 1970s [77]. More recent studies identified in this review complement this position, providing clear evidence for the sensitizing effect of transfusions. The level of sensitization was higher in transfused patients than in patients not receiving pre-transplant transfusion, with sensitization presumably resulting from factors such as previous pregnancy or prior transplantation. Additionally, the number of transfusions was correlated with the incidence of sensitization. Other studies have also reported that transfusions administered to patients with previous alloantigen exposure, such as pregnancies or transplants, and transfusions in other settings (heart transplants or hematological malignancies), often induce high sensitization [78]. Of note, universal leukoreduction has not decreased sensitization in patients to any significant degree [78]. Current data from transplant registries confirmed the sensitizing effect of transfusions, although these databases are limited regarding accurate assessment of the number of transfusions and by lack of subgroup analysis [27,73,74,76]. Overall, the results imply that avoiding transfusions can significantly decrease the incidence and degree of sensitization.

Numerous reports of the beneficial effects of blood transfusions on graft survival were published in the 1970s and early 1980s [79]. In contrast, articles published after 1984 reported a minor effect of blood transfusions, either beneficial or detrimental. Only 2 papers, dated 1987 and 1988, showed a more pronounced, non-significant, beneficial effect (17% and 19% better graft survival). However, a beneficial effect was observed when graft survival rates with no transfusions were low. The benefit was lower or non-existent when graft survival rates were higher, such as current rates. Thus, improvements in graft survival comparable with those attributed to the “transfusion effect” are now observed without transfusions, sparing patients the risks associated with pre-transplant transfusion. While a recent study reported a beneficial effect of pre-transplant transfusions with current immunosuppression protocols such as cyclosporine [80], the results from this review suggest that the graft outcome risk/benefit ratio has become too high to consider blood transfusions when they can be avoided. In addition, the beneficial effect of transfusions on graft survival implies that most patients transplanted remained weakly sensitized or non-sensitized after receiving transfusions; there are no reliable data to estimate the number of patients who became highly sensitized and were not able to receive a transplant following transfusion.

The majority of studies, as well as registry data, supported the concept that sensitization leads to higher rates of acute rejection, chronic rejection, antibody-mediated rejection, and graft loss. Two of the 6 studies that did not find an increase in the incidence of acute rejection with sensitization were restricted to evaluating B cell cross-match, although most studies on B cell cross-match reported that positive results led to decreased graft survival. Importantly, recent studies using solid phase techniques reported that it is not the sensitization per se that is harmful, but the presence of DSAs that lead to increased rejection and graft loss. A recent population-based study in over 2000 patients reported that DSAs increased graft rejection and were independent predictors of graft loss [81]. Since some studies have not found a correlation between DSAs and graft rejection, an emerging concept is that pre-transplant DSAs confer a risk of poorer outcomes, but the risk may be balanced by other factors in selected patients (live donor, increased immunosuppression, low overall sensitization) [41].

Sensitization also leads to increased waiting times. Although few papers have addressed this subject [24,49,75], this has been well documented in registry data [76]. Moderately sensitized patients’ wait times are close to non-sensitized patients’ wait times. However, highly sensitized patients continue to have excessive waiting times with the concomitant risk of death while waiting [76].

The AHRQ review [7,8] reported that pre-transplant transfusion resulted in a neutral to positive effect on graft rejection, graft survival, and patient survival compared with no transfusion. This review provides a historical analysis of pre-transplant transfusion, but few recent studies that involve current immunosuppressive medications, technologies and techniques were included in the analysis. In contrast to the AHRQ review, 2 recently published reviews reported results similar to our analysis indicating that blood transfusions are sensitizing, and HLA sensitization reduces graft survival and increases wait time for transplantation [82,83].

Overall, the evidence reviewed here suggests that blood transfusions can lead to high sensitization and negate the benefits of transplantation, therefore they should not be encouraged as first-line therapy of anemia. Alternatives to avoid transfusions should be considered whenever possible.

There are limitations with this review linked to limitations within each included study, and the limitations of registry data. Firstly, while most of the studies scored at least fair quality as assessed by the Downs and Black checklist [10], 6 studies were rated poor quality. Additionally, the data of interest to this review were not always the primary study endpoint, and may not be powered to detect differences between the subpopulations. Secondly, the detection techniques used in studies were different, making it difficult to directly compare results. The definition of sensitization varied, ranging from PRA > 5% to ≥ 30%, as well as the arbitrary cut-off levels for solid phase assays. There was also a lack of reporting on pregnancies or transplantations, which has important implications since previous alloantigen exposure can induce high levels of sensitization. Thirdly, information on immunosuppressant use was often suboptimal to allow differentiation of outcomes by the use or absence of such therapy. Furthermore, in some studies, immunosuppressive regimen use was routine and had an impact on transplantation outcomes. Fourthly, many studies presented data at multiple time points, with some conflicting results across time points, resulting in subjectivity in determining the overall outcome direction for each study (beneficial/detrimental). In addition, data were lacking in some areas, for instance in the assessment of pre-transplant transfusion and allosensitization on wait time to transplantation. The limitations of registry data are well reported, and in this study include no adjustment of confounding factors and the applicability of data from non-US registries to the US renal population.

## Conclusions

This review implicates pre-transplant transfusions in a number of untoward effects for potential transplant recipients. Avoiding transfusions whenever possible is now considered state-of-the-art practice for clinicians and surgeons of all specialties. Avoiding transfusions is important for candidates of kidney transplantation because of the risk of sensitization, with the concomitant possibility of longer wait times, becoming ineligible for a particular live donor, dying while on the waiting list, or having worse outcomes after transplantation.

## Abbreviations

AABB: American Association of Blood Banks; AHRQ: Agency for Healthcare Research and Quality; ANZDATA: Australia and New Zealand Dialysis and Transplant Registry; AST: American Society of Transplantation; CDC: Complement-dependent cytotoxicity; CDC-XM: Complement-dependent cytotoxicity cross-match; CsA: Cyclosporine A; CTS: Collaborative Transplant Study; DSA: Donor-specific antibody; DST: donor-specific transfusion; ELISA: enzyme-linked immunosorbant assay; ESA: Erythropoiesis-stimulating agent; FXM: Flow cytometry cross-match; HLA: human leukocyte antigen; HRSA: Health Resources and Services Administration; MeSH: Medical subject headings; MPC: Mean peak channel; NR: Not reported; OR: Odds ratio; PRA: Panel reactive antibodies; UNOS: United Network for Organ Sharing; USRDS: United States Renal Data System.

## Competing interests

JCS, JSB, and DJN are past paid consultants of Amgen Inc.

MB was an employee of HERON Evidence Development PVT at the time of study completion, which received funding from Amgen Inc. for the study.

MG and JP were employees of Amgen Inc. and had stock/stock options in Amgen Inc at the time of study.

## Authors’ contributions

JCS participated in data analysis and writing of the manuscript. JSB and DJN participated in data analysis. MB participated in research design and data analysis. MG and JP participated in research design, data analysis, and writing of the manuscript. All authors provided critical review of the manuscript and approved the final manuscript.

## Pre-publication history

The pre-publication history for this paper can be accessed here:

http://www.biomedcentral.com/1471-2369/14/217/prepub

## Supplementary Material

Additional file 1 **Example of Embase^®^ and MEDLINE^®^ search strategy for objective 1 (Association of pre-transplant transfusions with allosensitization).** Provides an example of the search strategy for objective 1. Click here for file
