# Supplementary material for: An update on the impact of pre-transplant transfusions and allosensitization on time to renal transplant and on allograft survival
Source: BMC Nephrol. 2013 Oct 10;14:217. doi: 10.1186/1471-2369-14-217 (PMC4125965; doi:10.1186/1471-2369-14-217)
Supplement: Additional file 1 — Example of Embase® and MEDLINE® search strategy for objective 1 (Association of pre-transplant transfusions with allosensitization). Provides an example of the search strategy for objective 1. [file 1471-2369-14-217-S1.pdf]

Example of Embase® and MEDLINE® search strategy for question 1 (Association of pre-transplant transfusions with allosensitization)

	Search term	Results
#1	'kidney'/de	231 545
#2	‘kidney transplantation’/exp	88 397
#3	renal:ab,ti	461 262
#4	kidney:ab,ti	278 367
#5	OR/1-4	738 570
#6	‘HLA system’/exp	16 791
#7	‘HLA typing’/exp	12 400
#8	('hla-dr' AND (mismatch* OR match*)):ab,ti	1390
#9	(hla NEAR/1 (matching OR typing)):ab,ti	4442
#10	'antibody'/de	100 059
#11	antibod*:ab,ti	684 329
#12	'alloantibody'/de	6362
#13	alloantibod*:ab,ti	3346
#14	'allosensiti?ation':ab,ti	252
#15	sensiti?ation:ab,ti	63 265
#16	sensiti?ed:ab,ti	36 389
#17	(‘flow beads' OR Luminex OR 'flow PRA' OR 'single antigen testing'):ab,ti	1768
#18	'histocompatibility'/de	17 250
#19	'histoincompatibility'/de	420
#20	Isoantibod*:ab,ti	371
#21	OR/6-20	829 811
#22	'blood transfusion'/de	73 042
#23	'blood component therapy'/de	1830
#24	'erythrocyte transfusion'/de	9356
#25	((blood OR 'red blood cells' OR erythrocyte*) NEAR/1 (transfusion* OR exchange OR infusion OR replacement OR retransfusion)):ab,ti	34 382
#26	(donor NEAR/2 transfusion*):ab,ti	1049
#27	(hemotherapy OR haemotherapy):ab,ti	660
#28	'transfusion therapy':ab,ti	1928
#29	(pretransplant* AND transfusion*):ab,ti	686
#30	OR/22-29	94 629
#31	#5 AND #21 AND #30	1593
